# Mapping the Landscape of Endemic Mycoses in India: Insights From a Systematic Review of Reported Cases Spanning 6 Decades (1960–2025)

**DOI:** 10.1093/ofid/ofag171

**Published:** 2026-03-21

**Authors:** Sidhya Choudhary, George R Thompson, Martin Hoenigl, S Nagarathna, Arghadip Samaddar

**Affiliations:** Department of Microbiology, Dr. Sampurnanand Medical College, Jodhpur, Rajasthan, India; Department of Internal Medicine, Division of Infectious Diseases, UC Davis Medical Center, Sacramento, California, USA; Division of Infectious Diseases, Department of Internal Medicine, Medical University of Graz, Graz, Austria; Translational Mycology Research Centre, Excellence Centre for Medical Mycology (ECMM), Department of Internal Medicine, Graz, Austria; Department of Neuromicrobiology, National Institute of Mental Health and Neuro Sciences, Bengaluru, Karnataka, India; Department of Neuromicrobiology, National Institute of Mental Health and Neuro Sciences, Bengaluru, Karnataka, India

**Keywords:** endemic mycoses, histoplasmosis, India, sporotrichosis, talaromycosis

## Abstract

**Background:**

Endemic mycoses are caused by fungi inhabiting specific ecological niches. These infections remain underreported due to diagnostic challenges and limited awareness. This study aims to provide a comprehensive synthesis of endemic mycoses in India, focusing on their distribution, clinical presentation, and outcomes.

**Method:**

A systematic literature search was conducted to identify Indian studies on proven endemic mycoses from 1960 to August 2025. Case reports, case series, and observational studies with laboratory-confirmed diagnosis of endemic mycoses were included, based on the European Organization for Research and Treatment of Cancer/Mycoses Study Group Education and Research Consortium (EORTC/MSGERC) criteria. Probable and possible cases were excluded. Demographic, clinical, diagnostic, treatment, and outcome data were extracted and analyzed using descriptive statistics.

**Results:**

A total of 230 studies comprising 893 patients were included. Histoplasmosis (399, 44.7%), sporotrichosis (386, 43.2%), and talaromycosis (87, 9.7%) were the most common infections. Cases were reported from 28 Indian states and union territories. Histoplasmosis was widely distributed, whereas sporotrichosis was concentrated in sub-Himalayan belt, and talaromycosis in north-eastern India. Reports increased sharply after 2000, peaking during 2011–2020. Histoplasmosis and talaromycosis predominantly affected males and caused disseminated disease, whereas sporotrichosis showed female predominance and manifested as cutaneous forms. Human immunodeficiency virus (HIV) infection was strongly associated with talaromycosis. Misdiagnosis as tuberculosis occurred in 7.6% of cases. Mortality was highest in histoplasmosis and coccidioidomycosis. In histoplasmosis, amphotericin B followed by itraconazole reduced mortality significantly.

**Conclusions:**

Endemic mycoses in India are increasingly recognized, with distinct epidemiological and clinical patterns. Persistent misdiagnosis and varied outcomes emphasize the need for improved diagnostics, clinician awareness, and optimized antifungal treatment strategies.

## Teaser

This systematic review synthesizes 893 proven endemic mycosis cases in India over 6 decades, showing increased reporting, frequent misdiagnosis as tuberculosis, pathogen-specific clinical patterns, and substantial mortality in histoplasmosis, highlighting major diagnostic gaps and the need for improved surveillance and treatment.

Endemic mycoses (histoplasmosis, sporotrichosis, talaromycosis, blastomycosis, coccidioidomycosis, emergomycosis, and paracoccidioidomycosis) are caused by a diverse group of fungi that inhabit specific ecological niches, resulting in geographically restricted distributions. These organisms are thermally dimorphic, existing as molds in the environment and converting to yeasts (or spherules) in human tissue. They are considered primary pathogens, capable of causing disease in both healthy and immunocompromised individuals [1, 2]. While often neglected in global health discourse, endemic mycoses pose significant diagnostic and therapeutic challenges due to their varied clinical presentations; overlapping symptoms with other infectious diseases, especially pulmonary tuberculosis (TB); and limited awareness among healthcare providers [2]. The global importance of endemic mycoses has recently been highlighted by the World Health Organization (WHO) fungal priority pathogens list, which classifies *Histoplasma capsulatum* as a high-priority pathogen, while *Coccidioides* spp. and *Paracoccidioides* spp. are designated as medium-priority pathogens, reflecting their substantial public health impact, diagnostic gaps, and limited availability of effective tools in resource-constrained settings [3]. Despite this recognition, these infections remain underdiagnosed and underreported in many endemic regions, including India.

In countries with diverse ecological and climatic zones, such as India, the burden and epidemiology of these infections are poorly characterized despite their potential to cause severe morbidity and mortality, particularly among immunocompromised populations [4]. India's vast geographical expanse, encompassing multiple climatic regions, provides a conducive environment for various endemic fungi. However, comprehensive data on the distribution, clinical features, and outcomes of endemic mycoses remain fragmented, often limited to anecdotal case reports, case series, and single-center observational studies. This knowledge gap is further compounded by the absence of a national registry, the lack of standardized hospital-based databases, and the nonexistence of mandatory notification or reporting systems for endemic mycoses in India. Consequently, the true incidence, geographic distribution, and temporal trends of these infections are likely grossly underestimated. Furthermore, with increasing prevalence of immunosuppressive conditions, such as human immunodeficiency virus (HIV)/ acquired immunodeficiency syndrome (AIDS), diabetes mellitus, and malignancies, along with growing use of immunosuppressive therapies and organ transplantation, the epidemiology of these fungal infections is evolving [5]. This changing landscape necessitates a detailed understanding of endemic mycoses to inform timely diagnosis, appropriate treatment, and effective public health interventions.

To date, no systematic assessment has been undertaken to map the distribution of endemic mycoses in India. A comprehensive overview is essential to identify regional hotspots where disease may be emerging or overlooked, assess risk factors, delineate clinical patterns, and evaluate diagnostic and therapeutic practices. This study aims to bridge this knowledge gap through a systematic review of published cases spanning 6 decades, providing the first large-scale synthesis of endemic mycoses in India.

## METHODS

The study was registered in PROSPERO online database (registration number CRD420251275178) and was conducted in accordance with the Preferred Reporting Items for Systematic Reviews and Meta-Analyses (PRISMA) 2020 guidelines [6]. The PRISMA checklists are provided as Supplementary File 1.

### Search Strategy and Selection Criteria

A comprehensive literature search was conducted in PubMed, Google Scholar, Embase, and Web of Science to identify studies published in English between 1960 and August 2025. The search strategy combined controlled vocabulary and free-text terms and included the following keywords: (“histoplasmosis” OR “sporotrichosis” OR “talaromycosis” OR “blastomycosis” OR “coccidioidomycosis” OR “emergomycosis” OR “paracoccidioidomycosis”) AND (“India” OR “Indian”). Gray literature and conference abstracts were excluded, and searches of clinical trial registries did not yield any relevant studies. Eligible publications included case reports, case series, cross-sectional studies, and retrospective or prospective observational studies that reported clinical, laboratory, or epidemiological data on histoplasmosis, sporotrichosis, talaromycosis, blastomycosis, coccidioidomycosis, emergomycosis, or paracoccidioidomycosis. Eligibility was restricted to proven cases of endemic mycoses originating from India that met the revised European Organization for Research and Treatment of Cancer/Mycoses Study Group Education and Research Consortium (EORTC/MSGERC) criteria for invasive fungal diseases [7]. Studies reporting probable or possible cases, as well as publications lacking primary patient-level data, relevant clinical information, or laboratory confirmation, were excluded.

### Selection Process and Data Extraction

Two reviewers (S. C. and A. S.) independently screened titles and abstracts to identify potentially eligible studies, retrieved full-text articles for assessment, and determined final study inclusion. Discrepancies at any stage were resolved through discussion and consultation with a third reviewer (M. H. or S. N.). Where data were missing or unclear, attempts were made to contact corresponding authors for clarification. For studies reporting individual patient-level data, summary statistics were generated, whereas aggregate data were extracted from studies reporting only study-level outcomes. Duplicate publications were identified through cross-referencing of author details, demographic information, and clinical characteristics and were excluded following consensus review.

### Risk of Bias

Given the heterogeneity of studies included, risk of bias was assessed qualitatively using the Joanna Briggs Institute (JBI) critical appraisal checklists. The appropriate JBI tools were applied for case reports, case series, and cohort studies [8]. Study quality was considered adequate when all checklist items were answered “yes”; however, studies deemed to be of inadequate quality were not excluded, in order to capture all available evidence on these rare infections.

### Data Analysis

Descriptive statistics were used to summarize demographic characteristics, clinical features, comorbidities, diagnostic modalities, treatment regimens, and outcomes across different types of endemic mycoses. Categorical variables were expressed as frequencies and percentages, and continuous variables were reported as medians with interquartile ranges (IQRs), given the nonnormal distribution of the data. Comparisons across disease categories were made for selected variables where appropriate. The Fisher–Freeman–Halton exact test was used for comparing proportions between the groups. Continuous variables were compared using the Kruskal–Wallis test. Subgroup analyses were performed to assess patterns of organ involvement, immune status (immunocompetent vs immunocompromised), and antifungal treatment regimens. Clinical features and imaging findings were also stratified by disease type. For rare mycoses with small sample sizes, data were primarily reported descriptively without inferential statistical testing due to limited power. No meta-analysis was attempted, owing to inconsistency and methodological heterogeneity of reviewed studies and differences in reporting. Associations between treatment strategies and mortality in histoplasmosis were evaluated using univariate logistic regression modeling, with mortality as the dependent variable. Odds ratios (ORs) with 95% confidence intervals (CIs) were calculated. All analyses were conducted using Statistical Package for the Social Sciences (SPSS) version 26 (IBM Corp., Armonk, NY, USA), and a 2-tailed *P*-value of <.05 was considered statistically significant.

## RESULTS

**Figure 1.**
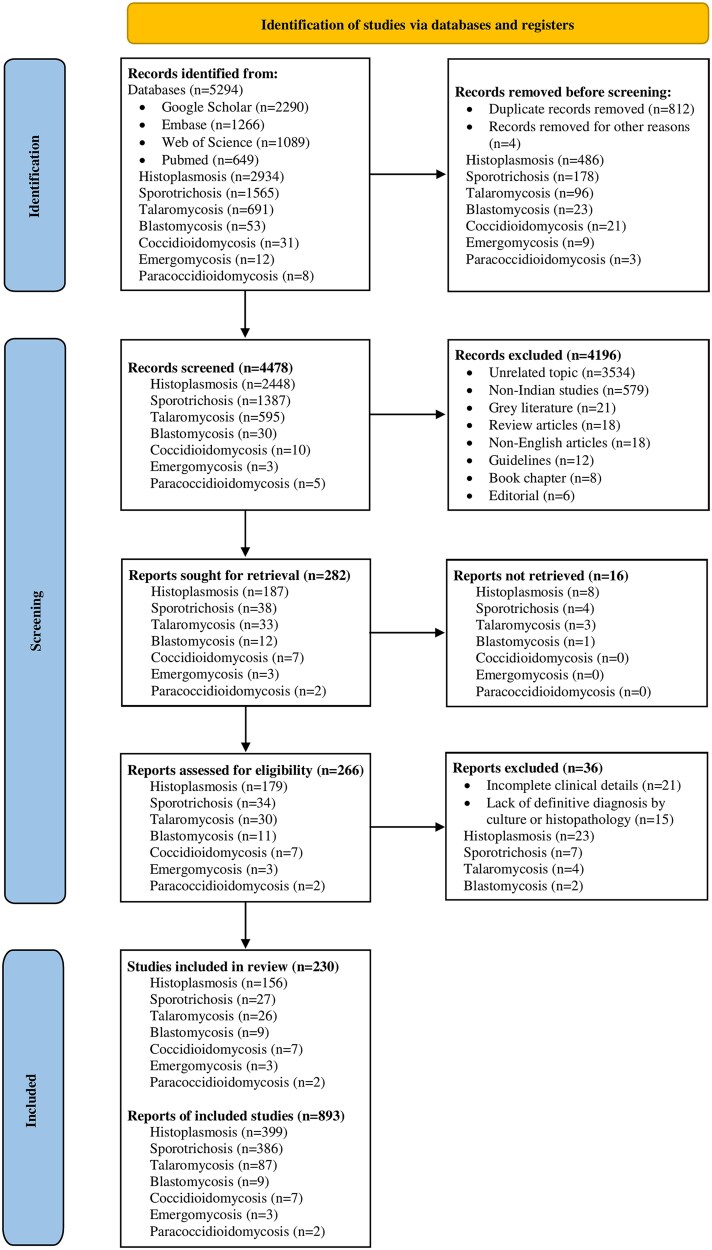
PRISMA diagram showing the study selection process. It depicts the total number of records identified through the literature and registry searches. Following the removal of duplicates, the remaining records were screened based on title and abstract. Records excluded at this stage were documented, with corresponding reasons for exclusion provided. Subsequently, full-text articles were assessed for eligibility, with the number of articles excluded and their reasons detailed. The flowchart concludes by presenting the total number of studies and cases included in this review.

A total of 5294 records related to endemic mycoses were identified from database searches. After removal of duplicates, 4478 records were screened, of which 4196 were excluded based on title and abstract review. Of the 282 records selected for full-text evaluation, manuscript could not be retrieved for 16 records, leaving 266 studies for full-text assessment; 36 were further excluded due to insufficient clinical details or lack of laboratory confirmation. Ultimately, 230 studies were included in the review, comprising 893 reported cases of proven endemic mycoses from India (Figure 1). The included studies were case reports (173/230, 75%), case series (30/230, 13%), and retrospective cohort studies (27/230, 12%), with 84.3% of studies judged to be of acceptable quality. The citation, characteristics, and risk of bias for each study are summarized in Supplementary Table 1.

**Figure 2.**
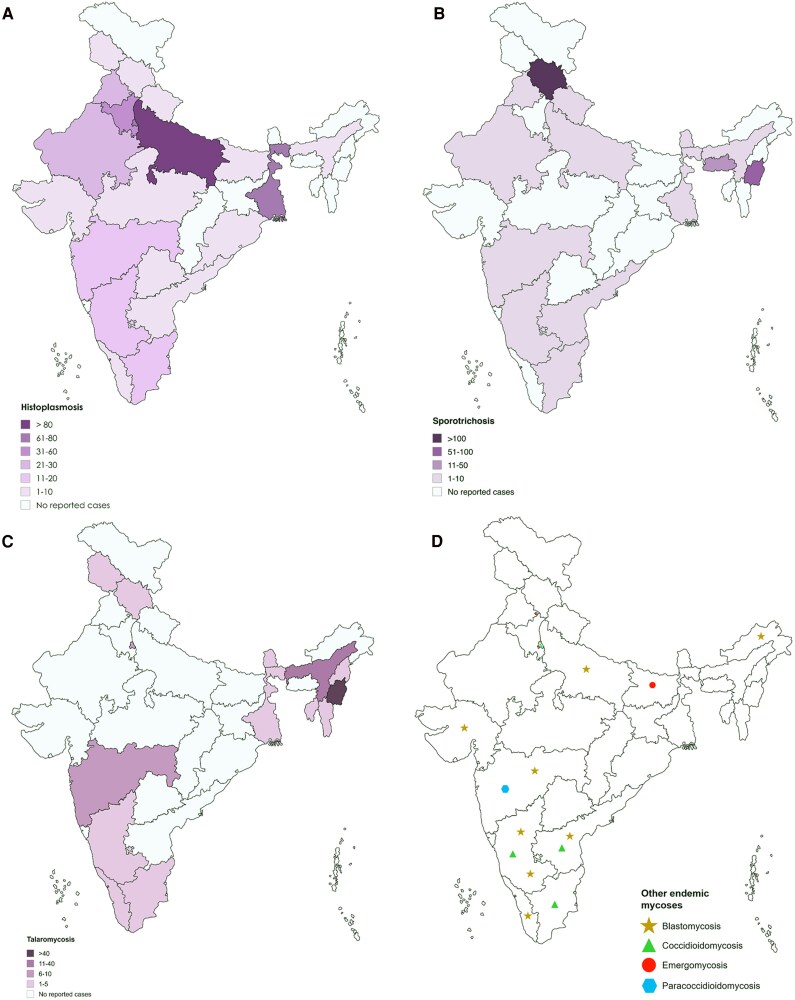
Map of India showing total cases of endemic mycoses reported from various states between 1960 and August 2025. *A*, Histoplasmosis cases were reported from Uttar Pradesh (n = 94), West Bengal (n = 71), New Delhi (n = 61), Haryana (n = 33), Rajasthan (n = 27), Punjab (n = 25), Maharashtra (n = 20), Tamil Nadu (n = 14), Karnataka (n = 11), Chandigarh and Gujarat (n = 7, each), Himachal Pradesh and Kerala (n = 6, each), Bihar and Uttarakhand (n = 4, each), Madhya Pradesh (n = 3), Orissa (n = 2), and Andhra Pradesh, Assam, Telangana, and Jammu and Kashmir (n = 1, each). *B*, Sporotrichosis cases were reported from Himachal Pradesh (n = 259), Manipur (n = 74), Meghalaya (n = 16), Karnataka (n = 6), Maharashtra (n = 4), Sikkim (n = 3), and Andhra Pradesh, Punjab, Rajasthan, Tamil Nadu, Uttar Pradesh, Uttarakhand, West Bengal, and New Delhi (n = 1, each). *C*, Talaromycosis cases were reported from Manipur (n = 432), Assam (n = 11), New Delhi (n = 10), Maharashtra (n = 7), Sikkim (n = 5), Himachal Pradesh (n = 2), and Karnataka, Kerala, Nagaland, Tamil Nadu, Tripura, West Bengal, and Jammu and Kashmir (n = 1, each). *D*, Other endemic mycoses include blastomycosis (n = 9), coccidioidomycosis (n = 8), emergomycosis (n = 3), and paracoccidioidomycosis (n = 2). Blastomycosis cases were reported from Karnataka (n = 2) and Andhra Pradesh, Arunachal Pradesh, Gujarat, Kerala, Maharashtra, Uttar Pradesh, and New Delhi (n = 1, each). Coccidioidomycosis was reported from New Delhi (n = 4), Karnataka (n = 2), and Andhra Pradesh and Tamil Nadu (n = 1, each). Emergomycosis was reported from Bihar, Chandigarh, and New Delhi (n = 1, each). Paracoccidioidomycosis was reported from Maharashtra and New Delhi (n = 1, each).

The majority of cases were histoplasmosis (399/893, 44.7%), sporotrichosis (386/893, 43.2%), and talaromycosis (87/893, 9.7%), while blastomycosis, coccidioidomycosis, emergomycosis, and paracoccidioidomycosis accounted for less than 2% combined (Table 1). A total of 28 Indian states and union territories reported cases of endemic mycoses (Figure 2). Histoplasmosis was the most widely distributed, reported from 21 states, with the highest case counts in Uttar Pradesh (n = 94), West Bengal (n = 71), New Delhi (n = 61), and Haryana (n = 33). Sporotrichosis was notably concentrated in Himachal Pradesh (n = 259) and Manipur (n = 74), together accounting for 86.3% of all reported cases. Talaromycosis cases were predominantly reported from Manipur (n = 43). Blastomycosis was rare, reported from a limited number of states, including Karnataka, New Delhi, and Andhra Pradesh. Coccidioidomycosis was documented in 5 locations, with New Delhi reporting the highest count (n = 4). Emergomycosis was reported from Chandigarh, New Delhi, and Bihar and paracoccidioidomycosis from New Delhi and Maharashtra.

**Table 1.** Characteristics of Reported Cases of Proven Endemic Mycoses in India (1960–2025)

Parameters	N/Total Patients (%)^a^						
	Histoplasmosis	Sporotrichosis	Talaromycosis	Blastomycosis	Coccidioidomycosis	Emergomycosis	Paracoccidioidomycosis
N/total endemic mycosis cases (%)	399/893 (44.7)	386/893 (43.3)	87/893 (9.7)	9/893 (1)	7/893 (0.8)	3/893 (0.3)	2/893 (0.2)
Male	328/380 (86.3)	161/383 (41.7)	71/87 (81.6)	6/9 (66.7)	7/7 (100)	1/3 (33.3)	2/2 (100)
Female	52/380 (13.7)	222/383 (58.3)	16/87 (18.4)	3/9 (33.3)	0	2/3 (66.7)	0
Age, median (IQR)	49 (3–84)	45.5 (1.5–86)	31 (3–66)	37 (6–53)	31 (22–65)	38 (27–73)	24.5 (14–35)
Clinical features							
Fever	243/399 (61)	1/386 (0.3)	63/87 (72.4)	6/9 (66.7)	7/7 (100)	2/3 (66.7)	1/2 (50)
Weight loss	228/399 (57)	0	66/87 (76)	2/9 (22.2)	2/7 (28.6)	1/3 (33.3)	0
Anorexia	112/399 (28)	0	1/87 (1.1)	2/9 (22.2)	0	0	0
Fatigue	92/399 (23)	0	0	0	0	0	0
Generalized weakness	39/399 (9.8)	0	35/87 (40.2)	0	0	0	0
Cough	78/399 (19.5)	1/386 (0.3)	12/87 (13.8)	4/9 (44.4)	4/7 (57.1)	1/3 (33.3)	0
Dyspnea	51/399 (12.8)	0	0	1/9 (11.1)	0	2/3 (66.7)	0
Skin lesions	82/399 (20.6)	384/386 (99.5)	54/87 (62.1)	4/9 (44.4)	1/7 (14.3)	2/3 (66.7)	1/2 (50)
Cutaneous sinuses	0	10/386 (2.6)	0	3/9 (33.3)	2/7 (28.6)	0	0
Hyperpigmentation	51/399 (12.8)	0	1/87 (1.1)	0	0	0	0
Oral ulcers	24/399 (6)	0	0	0	0	0	0
Tongue ulcer	26/399 (6.5)	0	0	0	0	0	0
Palatal ulcer	19/399 (4.8)	0	0	0	0	0	0
Dysphagia	17/399 (4.3)	0	0	0	0	0	0
Chest pain	15/399 (3.8)	0	0	0	0	0	0
Hoarseness	10/399 (2.5)	0	0	0	0	0	0
Abdominal pain	57/399 (14.3)	0	2/87 (2.3)	0	0	0	0
Diarrhea	10/399 (2.5)	0	0	0	0	0	0
Vomiting	7/399 (1.7)	0	0	0	0	0	0
Adrenal insufficiency	30/399 (7.5)	0	0	0	0	0	0
CNS symptoms	11/399 (2.7)	0	0	0	1/7 (14.3)	0	1/2 (50)
Others	17/399 (4.3)	0	2/87 (2.3)	2/9 (22.2)	1/7 (14.3)	0	0
H/o ATT	34/399 (8.5)	0	19/87 (21.8)	3/9 (33.3)	3/7 (42.8)	0	0
Travel history	7/399 (1.7)	0	0	3/9 (33.3)	6/7 (85.7)	0	0
H/o trauma	0	184/386 (47.7)	1/87 (1.1)	0	0	0	0
IVDU	0	0	32/87 (36.8)	0	0	0	0
System involved							
Primary pulmonary	12/399 (3)	1/386 (0.3)	12/87 (13.8)	1/9 (11.1)	3/7 (42.8)	1/3 (33.3)	0
Primary cutaneous	18/399 (4.5)	0	16/87 (18.4)	2/9 (22.2)	2/7 (28.6)	2/3 (66.7)	1/2 (50)
Mucocutaneous	41/399 (10.3)	0	2/87 (2.3)	1/9 (11.1)	0	0	0
Disseminated	316/399 (79.2)	1/386 (0.3)	57/87 (65.5)	4/9 (44.4)	1/7 (14.3)	0	0
Genitourinary	4/399 (1)	0	0	0	0	0	0
Ocular	3/399 (0.7)	2/386 (0.5)	0	0	0	0	0
CNS	2/399 (0.5)	0	0	1/9 (11.1)	0	0	1/2 (50)
Osteoarticular	2/399 (0.5)	3/386 (0.8)	0	0	1/7 (14.3)	0	0
CVS	1/399 (0.3)	0	0	0	0	0	0
Lymphocutaneous	-	218/386 (56.5)	-	-	-	-	-
Fixed cutaneous	-	161/386 (41.7)	-	-	-	-	-
Anatomical site							
Head and neck	5/399 (1.2)	74/386 (19.2)	14/31 (45.2)	3/9 (33.3)	1/7 (14.3)	2/3 (66.7)	2/2 (100)
Oral cavity	41/399 (10.3)	0	1/31 (3.2)	0	0	0	0
Upper limb	17/399 (4.3)	205/386 (53.1)	6/31 (19.4)	1/9 (11.1)	1/7 (14.3)	1/3 (33.3)	0
Lower limb	7/399 (1.8)	89/386 (23)	1/31 (3.2)	3/9 (33.3)	1/7 (14.3)	1/3 (33.3)	0
Thorax	12/399 (3)	15/386 (3.9)	2/31 (6.5)	2/9 (22.2)	3/7 (42.8)	3/3 (100)	0
Multisystem	316/399 (79.2)	1/386 (0.3)	4/31 (12.9)	1/9 (11.1)	1/7 (14.3)	0	0
Others^b^	7/399 (1.8)	2/386 (0.5)	1/31 (3.2)	0	0	0	0
Comorbidities							
Type 2 DM	78/399 (19.5)	2/28 (7.1)	2/77 (2.6)	2/9 (22.2)	3/7 (42.8)	1/3 (33.3)	0
HIV/AIDS	45/399 (11.3)	3/28 (10.7)	74/77 (96.1)	0	1/7 (14.3)	2/3 (66.7)	0
Immunosuppressive drugs^c^	34/399 (8.5)	0	0	2	0	1	0
Solid organ transplant	15/399 (3.8)	0	1/77 (1.3)	1/9 (11.1)	0	1/3 (33.3)	0
Autoimmune disorders	10/399 (2.5)	0	0	0	0	0	0
Primary immunodeficiency disorders	7/399 (1.7)	0	0	1/9 (11.1)	0	0	0
Hypertension	7/399 (1.7)	1/28 (3.6)	0	0	2/7 (28.6)	1/3 (33.3)	0
Hematologic malignancy	5/399 (1.2)	0	0	0	0	0	0
CKD	5/399 (1.2)	0	0	0	1/7 (14.3)	0	0
CLD	4/399 (1)	0	0	0	0	0	0
COPD	3/399 (0.7)	1/28 (3.6)	0	1/9 (11.1)	2/7 (28.6)	0	0
Inflammatory bowel disease	3/399 (0.7)	0	0	0	0	0	0
Cardiac disease	3/399 (0.7)	0	0	0	0	1/3 (33.3)	0
Hepatitis B	1/399 (0.2)	0	0	0	0	0	0
Hepatitis C	1/399 (0.2)	0	0	0	0	0	0
Hypothyroidism	1/399 (0.2)	4/28 (14.3)	0	0	0	1/3 (33.3)	0
Misdiagnosed as TB	48/399 (12)	9/386 (2.3)	7/87 (8)	2/9 (22.2)	2/7 (28.6)	0	0
Coinfection							
Fungal^d^	0	0	5/87 (5.7)	0	0	0	0
Viral^e^	1/399 (0.2)	0	6/87 (6.9)	0	0	0	0
Parasitic^f^	3/399 (0.7)	0	0	0	0	0	0
Immune status							
Immunocompetent	269/355 (75.8)	173/176 (98.3)	2/77 (2.6)	7/9 (77.8)	5/7 (71.4)	0	2/2 (100)
Immunocompromised	86/355 (24.2)	3/176 (1.7)	75/77 (97.4)	2/9 (22.2)	2/7 (28.6)	3/3 (100)	0
Imaging findings							
Hepatosplenomegaly	130/399 (32.6)	0	26/74 (35.1)	1/9 (11.1)	0	0	0
Lymphadenopathy	118/399 (29.6)	0	22/74 (29.7)	1/9 (11.1)	2/7 (28.6)	3/3 (100)	0
Adrenal mass	202/399 (50.6)	0	0	1/9 (11.1)	0	0	0
Bilateral	151/399 (37.8)	-	-	1/9 (11.1)	-	-	-
Unilateral	51/399 (12.8)	-	-	0	-	-	-
Lung lesions	49/191 (25.6)	2/14 (14.3)	10/74 (13.5)	5/9 (55.5)	3/7 (42.8)	3/3 (100)	0
Pleural effusion	9/191 (4.7)	0	1/74 (1.4)	0	1/7 (14.3)	1/3 (33.3)	0
CNS lesions	2/18 (11.1)	0	0	1/9 (11.1)	1/7 (14.3)	0	1/2 (50)
GI lesions	11/399 (2.7)	0	3/74 (4)	0	0	0	0
Others^g^	9/399 (2.3)	1/14 (7.1)	0	1/9 (11.1)	2/7 (28.6)	0	0
Sample type							
Blood culture	4/399 (1)	0	39/87 (44.8)	0	1/7 (14.3)	0	0
Adrenal biopsy	178/399 (44.6)	0	0	1/9 (11.1)	0	0	0
Skin biopsy	49/399 (12.3)	249/386 (64.5)	57/87 (65.5)	3/9 (33.3)	1/7 (14.3)	2/3 (66.7)	1/2 (50)
BM aspirate	55/399 (13.8)	0	2/87 (2.3)	0	1/7 (14.3)	0	0
LN aspirate	37/399 (9.3)	0	15/87 (17.2)	2/9 (22.2)	2/7 (28.6)	0	0
Pus	0	131/386 (34)	0	1/9 (11.1)	0	0	0
Tongue biopsy	18/399 (4.5)	0	0	0	0	0	0
Palatal biopsy	12/399 (3)	0	0	0	0	0	0
Mucosal biopsy	15/399 (3.8)	1/386 (0.3)	3/87 (3.4)	0	0	0	0
GI biopsy	20/399 (5)	0	1/87 (1.1)	0	0	0	0
Liver biopsy	6/399 (1.5)	0	0	0	0	0	0
Splenic aspirate	4/399 (1)	0	0	0	0	0	0
Sputum	0	1/386 (0.3)	10/87 (11.5)	1/9 (11.1)	0	0	0
Others^h^	19/399 (4.8)	4/386 (1)	8/87 (9.2)	1/9 (11.1)	3/7 (42.8)	2/3 (66.7)	1/2 (50)
Diagnosis							
Direct microscopy	0	6/365 (1.6)	1/87 (1.1)	0	1/7 (14.3)	1/3 (33.3)	1/2 (50)
HPE	286/396 (72.2)	131/365 (35.9)	30/87 (34.5)	4/9 (44.4)	3/7 (42.8)	2/3 (66.7)	2/2 (100)
Culture	9/396 (2.3)	147/365 (40.3)	44/87 (50.6)	1/9 (11.1)	3/7 (42.8)	3/3 (100)	0
HPE + culture	101/396 (25.5)	86/365 (23.6)	13/87 (14.9)	4/9 (44.4)	0	2/3 (66.7)	0
Antigen positive	6/6 (100)	-	-	-	-	-	-
CF test positive	3/3 (100)	-	-	-	-	-	-
Antifungal drugs^i^							
AmB alone	57/275 (20.7)	0	3/56 (5.4)	1/8 (12.5)	0	0	0
ITZ alone	87/275 (31.6)	28/308 (9.1)	45/56 (80.3)	7/8 (87.5)	0	0	1/1 (100)
FLC alone	3/275 (1.1)	3/308 (1)	1/56 (1.8)	0	3/6 (50)	0	0
AmB f/b ITZ	122/275 (44.4)	1/308 (0.3)	5/56 (8.9)	0	2/6 (33.3)	3/3 (100)	0
AmB f/b FLC	0	0	2/56 (3.6)	0	1/6 (16.7)	0	0
SSKI	0	247/308 (80.2)	0	0	0	0	0
SSKI f/b ITZ	0	27/308 (8.8)	0	0	0	0	0
Others^j^	6/275 (2.2)	2/308 (0.6)	0	0	1/6 (16.7)	0	0
Median treatment duration (IQR) days^k^	194 (2–1460)	120 (60–480)	-	180 (180–543)	30 (7–365)	379 (44–379)	180
Outcome							
Survived	235/288 (81.6)	176/177 (99.4)	46/50 (92)	9/9 (100)	4/6 (66.7)	2/3 (66.7)	0
Immunocompetent	164/235 (69.8)	15/176 (8.5)	3/46 (6.5)	7/9 (77.8)	4/4 (100)	0	-
Immunocompromised	60/235 (25.5)	1/176 (0.6)	43/46 (93.5)	2/9 (22.2)	0	2/2 (100)	-
Immune status unknown	11/235 (4.7)	160/176 (91)	0	0	0	0	…
Died	53/288 (18.4)	1/177 (0.6)	4/50 (8)	0	2/6 (33.3)	1/3 (33.3)	1/1 (100)
Immunocompetent	35/53 (66)	0	0	-	1/2 (50)	0	1/1 (100)
Immunocompromised	17/53 (32.1)	1/1 (100)	4/4 (100)	-	1/2 (50)	1/1 (100)	0
Immune status unknown	1/53 (1.9)	0	0	-	0	0	0

Abbreviations: AIDS, acquired immunodeficiency syndrome; AmB, amphotericin B; ATT, antitubercular therapy; BM, bone marrow; CF, complement fixation; CKD, chronic kidney disease; CLD, chronic liver disease; CNS, central nervous system; COPD, chronic obstructive pulmonary disease; CVS, cardiovascular system; DM, diabetes mellitus; FLC, fluconazole; GI, gastrointestinal; HIV, human immunodeficiency virus; HPE, histopathological examination; IQR, interquartile range; ITZ, itraconazole; IVDU, intravenous drug users; LN, lymph node; SSKI, saturated solution of potassium iodide; TB, tuberculosis.

^a^Denominator for each parameter indicates the number of cases with available data.

^b^Other sites for histoplasmosis include the penis (n = 3), eye (n = 3), and knee joint (n = 1); sporotrichosis, bone (n = 1) and genital (n = 1); and talaromycosis, genital (n = 1).

^c^Immunosuppressive drugs in histoplasmosis patients include steroids (n = 26), tacrolimus (n = 15), mycophenolate mofetil (n = 9), cyclosporine (n = 7), azathioprine (n = 5), rituximab (n = 4), and cyclophosphamide (n = 3); blastomycosis, steroids (n = 2) and tacrolimus (n = 1); and emergomycosis, prednisolone (n = 1), tacrolimus (n = 1), and mycophenolate mofetil (n = 1).

^d^Fungal coinfections in talaromycosis include oral candidiasis (n = 4) and cryptococcal meningitis (n = 1).

^e^Viral coinfections in histoplasmosis include varicella zoster virus (n = 1) and talaromycosis, Epstein–Barr virus (n = 3), cytomegalovirus (n = 1), varicella zoster virus (n = 1), and herpes simplex virus (n = 1).

^f^Parasitic coinfection in histoplasmosis includes leishmaniasis (n = 3).

^g^Other imaging findings in histoplasmosis include ascites (n = 6), subpleural nodule (n = 1), prostatomegaly (n = 1), enlarged epididymis (n = 1), vertebral abscess (n = 1), and renal artery stenosis (n = 1); sporotrichosis, maxillary sinus space-occupying lesion (n = 1); blastomycosis, extraconal orbital mass (n = 1); and coccidioidomycosis, lytic bone lesions (n = 2).

^h^Other sample types in histoplasmosis include laryngeal biopsy (n = 7), penile biopsy (n = 3), brain biopsy (n = 2), bronchoalveolar lavage fluid (BALF) (n = 2), pleural biopsy (n = 1), testicular biopsy (n = 1), bone biopsy (n = 1), pleural fluid (n = 1), and seminal fluid (n = 1); sporotrichosis, corneal scraping (n = 2), bone biopsy (n = 1), and postmortem lung biopsy (n = 1); talaromycosis, nail clipping (6) and lung biopsy (n = 2); blastomycosis, brain biopsy (n = 1); coccidioidomycosis, lung biopsy (n = 2) and BALF (n = 1); emergomycosis, lung biopsy (n = 1) and BALF (n = 1); and paracoccidioidomycosis, brain biopsy (n = 1).

^i^Six histoplasmosis and 1 talaromycosis patients succumbed before appropriate antifungal therapy could be instituted.

^j^Other treatments used for histoplasmosis include topical nystatin (n = 3), voriconazole (n = 1), ketoconazole (n = 1), and hamycin (n = 1); sporotrichosis, terbinafine (n = 1) and thermotherapy (n = 1); and coccidioidomycosis, 5-flucytosine (n = 1).

^k^For patients treated with antifungals, the duration of therapy was unknown for 195 histoplasmosis, 120 sporotrichosis, and 2 coccidioidomycosis cases. Treatment duration was unavailable for all talaromycosis cases.

**Figure 3.**
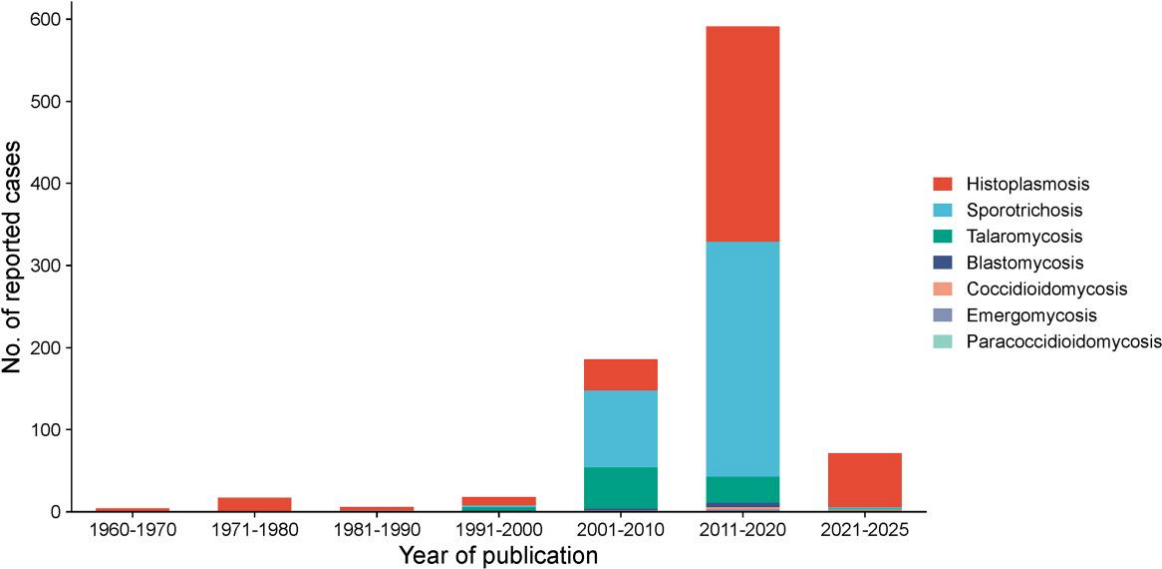
Decadal distribution of reported cases of endemic mycoses in India from 1960 to August 2025. Stacked bars show the decade-wise number of cases attributed to each endemic mycosis, highlighting a marked increase in reported cases after 2000, particularly during 2011–2020.

Analysis of endemic mycoses from 1960 to August 2025 demonstrated a substantial increase in both the number and diversity of reported cases over time, with a pronounced escalation after 2000 and a peak during 2011–2020 (Figure 3). Histoplasmosis showed the largest rise, increasing from 38 reports in 2001–2010 to 262 in 2011–2020, compared with relatively low and stable counts before 2000 (4–16 per decade). Sporotrichosis was rarely reported prior to 2000 (≤2 per decade) but increased sharply to 94 cases in 2001–2010 and 286 in 2011–2020, representing the highest decade-specific burden among all mycoses. Talaromycosis emerged later, rising from 4 reports in 1991–2000 to 50 in 2001–2010, followed by a modest decline to 32 in 2011–2020. Blastomycosis and coccidioidomycosis remained infrequently reported but increased after 2000 (blastomycosis, 2–5; coccidioidomycosis, 2–3, from 2001–2010 to 2011–2020). Emergomycosis and paracoccidioidomycosis were reported only in the most recent decades (2011–2025). Lower counts in 2021–2025 likely reflect the shorter observation period.

Demographic details and clinical characteristics of proven endemic mycoses in India are shown in Table 1. Male predominance was noted in histoplasmosis (328/380, 86.3%) and talaromycosis (71/87, 81.6%), while sporotrichosis showed a female preponderance (222/383, 58.3%) (*P* < .001). All reported cases of coccidioidomycosis and paracoccidioidomycosis occurred in males. The median age at presentation varied significantly across infections (Kruskal–Wallis, *P* < .001). Histoplasmosis (49 years; IQR, 3–84) and sporotrichosis (45.5 years; IQR, 1.5–86) primarily affected middle-aged individuals, whereas talaromycosis (31 years; IQR, 3–66) and coccidioidomycosis (31 years; IQR, 22–65) occurred in relatively younger patients. Paracoccidioidomycosis was observed in the youngest age group overall. Occupational history and exposure records were available for 64 histoplasmosis and 320 sporotrichosis cases. Notably, 12 individuals with histoplasmosis and 313 with sporotrichosis were engaged in agriculture-related occupations, while 13 histoplasmosis patients reported exposure to birds. Occupational exposure data were either unavailable or not identified as relevant for other endemic mycoses. Seven patients with histoplasmosis, 3 with blastomycosis, and 6 with coccidioidomycosis had a history of travel to endemic regions, particularly the Great Lakes area of the United States.

**Figure 4.**
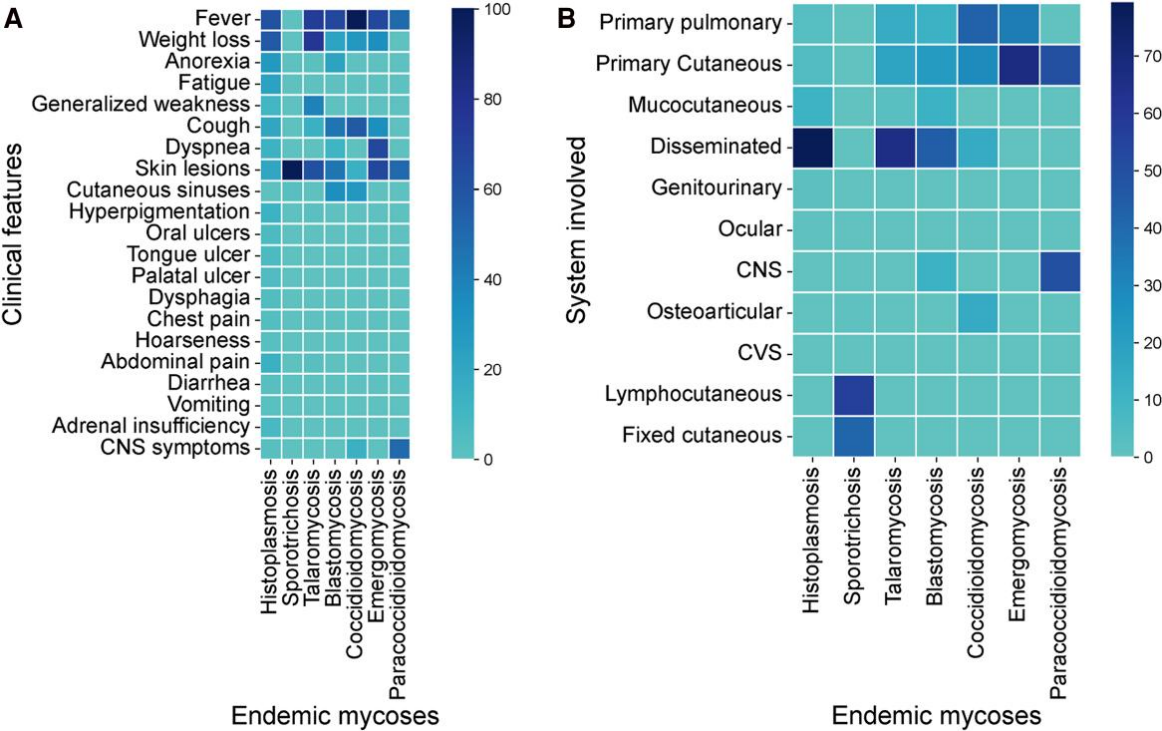
Heatmaps showing the distribution of clinical features (left) and organ/system involvement (right) across endemic mycoses. Color intensity represents the proportion of reported cases (%) in which a given feature or system involvement was observed. The scale is based on percentage frequency (proportion of affected patients). Abbreviations: CNS, central nervous system; CVS, cardiovascular system.

Fever was common in histoplasmosis (243/399, 61%), talaromycosis (63/87, 72.4%), blastomycosis (6/9, 66.7%), and coccidioidomycosis (7/7, 100%), but rare in sporotrichosis (1/386, .3%). Weight loss was significantly more frequent in talaromycosis (66/87, 76%) than in other infections (*P* < .001). Skin lesions were nearly universal in sporotrichosis (384/386, 99.5%) and frequent in emergomycosis (2/3, 66.7%), talaromycosis (54/87, 62.1%), and blastomycosis (4/9, 44.4%). Oral lesions, tongue and palatal ulcers, and adrenal involvement were almost exclusive to histoplasmosis. Cutaneous draining sinuses were seen in sporotrichosis, blastomycosis, and coccidioidomycosis. Respiratory symptoms, including cough and dyspnea, were more frequent in blastomycosis and coccidioidomycosis. Central nervous system (CNS) involvement was noted in histoplasmosis, emergomycosis, and paracoccidioidomycosis (Figure 4*A*). Disseminated disease was most frequent in histoplasmosis (316/399, 79.2%), followed by talaromycosis (57/87, 65.5%) and blastomycosis (4/9, 44.4%). In sporotrichosis, lymphocutaneous (218/386, 56.5%) and fixed cutaneous (161/386, 41.7%) forms were predominant (Figure 4*B*).

Underlying comorbidities, disease severity, and outcomes differed significantly across endemic mycoses (Table 2). Type 2 diabetes mellitus was more frequently observed among patients with histoplasmosis and coccidioidomycosis compared with other infections (*P* < .0001). HIV infection and immunocompromised status were overwhelmingly high among patients with talaromycosis (*P* < .0001), whereas sporotrichosis occurred predominantly in immunocompetent hosts. The likelihood of disseminated disease varied markedly by pathogen, being most frequent in histoplasmosis and talaromycosis and rare in sporotrichosis and coccidioidomycosis (*P* < .0001). Intravenous drug use was a significant risk factor for HIV-associated talaromycosis (32/87, 36.8%) (*P* < .001) (Table 1).

**Table 2.** Comparison of Selected Clinical Predictors and Outcomes Across Major Endemic Mycoses in India

Variable	N/Total Cases (%)					*P*-Value^a^
	Histoplasmosis	Sporotrichosis	Talaromycosis	Blastomycosis	Coccidioidomycosis	
Type 2 DM	78/399 (19.5)	2/28 (7.1)	2/77 (2.6)	2/9 (22.2)	3/7 (42.8)	**.0003**
HIV/AIDS	45/399 (11.3)	3/28 (10.7)	74/77 (96.1)	0	1/7 (14.3)	**<.0001**
Immunocompromised status	86/355 (24.2)	3/176 (1.7)	75/77 (97.4)	2/9 (22.2)	2/7 (28.6)	**<.0001**
Disseminated disease	316/399 (79.2)	1/386 (0.3)	57/87 (65.5)	4/9 (44.4)	1/7 (14.3)	**<.0001**
Mortality	53/288 (18.4)	1/177 (0.6)	4/50 (8)	0	2/6 (33.3)	**.0046**

Comparisons are limited to histoplasmosis, sporotrichosis, talaromycosis, blastomycosis, and coccidioidomycosis.

The Fisher–Freeman–Halton exact test shows significant heterogeneity in the distribution of Type 2 DM, HIV/AIDS, immunocompromised status, disseminated disease, and mortality across endemic mycoses (all *P* < .05), with particularly strong differences observed for HIV/AIDS, immunocompromised status, and disseminated disease.

Abbreviations: AIDS, acquired immunodeficiency syndrome; DM, diabetes mellitus; HIV, human immunodeficiency virus.

^a^Comparisons of clinical and epidemiological variables across disease groups were exploratory and descriptive; reported *P*-values indicate differences in distributions between groups and do not imply causal relationships. The values in bold denote statistical significance (*P* < .05).

Adrenal masses were characteristic of histoplasmosis (202/399, 50.6%) but uncommon in other infections. Hepatosplenomegaly and lymphadenopathy were frequent in histoplasmosis and talaromycosis but rare in others (*P* < .001). Lung lesions were most frequent in blastomycosis (5/9, 55.5%), coccidioidomycosis (3/7, 42.8%), and emergomycosis (100%) (*P* < .001) (Table 1).

Adrenal biopsy was the most frequently performed diagnostic procedure for histoplasmosis (178/399, 44.6%), whereas skin biopsy was the predominant method in sporotrichosis (249/386, 64.5%) and talaromycosis (57/87, 65.5%). Histopathological examination served as the primary diagnostic modality in histoplasmosis (286/396, 72.2%) and sporotrichosis (131/365, 36%), while culture positivity was highest in talaromycosis (44/87, 50.6%). Direct microscopy contributed to diagnosis in a minority of cases (<3%). Antigen detection and serological tests were performed only in a small number of histoplasmosis cases. A total of 68 (7.6%) cases were misdiagnosed as TB, including 48 (12%) histoplasmosis, 9 (2.3%) sporotrichosis, 7 (8%) talaromycosis, 2 (22.2%) blastomycosis, and 2 (28.6%) coccidioidomycosis cases (Table 1).

Antifungal treatment varied by infection type (Table 1). Induction therapy with intravenous amphotericin B (AmB) deoxycholate, followed by maintenance with oral itraconazole (ITZ) was commonly used in histoplasmosis (122/275, 44.4%) and emergomycosis (3/3, 100%). Saturated solution of potassium iodide (SSKI) ± ITZ was the standard treatment for cutaneous sporotrichosis. Itraconazole alone was the primary treatment in talaromycosis (45/56, 80.3%) and blastomycosis (7/8, 87.5%). Median treatment duration varied significantly (*P* < .001), longest for emergomycosis (379 days; IQR, 44–379) and shortest for coccidioidomycosis (30 days; IQR, 7–365).

Mortality differed significantly across endemic mycoses, with higher case fatality observed in histoplasmosis and coccidioidomycosis and minimal to no mortality in sporotrichosis and blastomycosis (*P* = .0046) (Table 2). Among histoplasmosis patients with follow-up data, 19% died during treatment. On univariate analysis, demographic factors, underlying comorbidities, and disease extent were not significantly associated with mortality in histoplasmosis (Table 3). However, antifungal treatment strategy was strongly associated with outcome. Receipt of AmB monotherapy was associated with increased odds of death (OR, 5.77; 95% CI, 2.66–12.5; *P* < .0001). The most pronounced survival benefit was observed among patients treated with AmB followed by ITZ therapy, which was independently associated with substantially reduced odds of mortality (OR, 0.19; 95% CI, 0.07–0.51; *P* = .001) compared with other treatment approaches.

**Table 3.** Predictors of Mortality in Histoplasmosis based on Univariate Logistic Regression Analysis

Factor	Survived	Died	OR	95% CI	*P*-Value
Age ≥ 45 y	150	33	0.92	.49–1.71	.80
Male gender	203	47	1.23	.49–3.12	.66
HIV/AIDS	28	10	1.72	.78–3.8	.18
Immunocompromised	60	17	1.33	.69–2.54	.39
Disseminated disease	184	45	1.56	.69–3.52	.28
Adrenal involvement	115	20	0.63	.34–1.17	.14
Pulmonary involvement	39	6	1.32	.46–3.76	.60
Type 2 DM	55	8	0.57	.25–1.28	.17
Immunosuppressive	26	6	1.02	.40–2.63	.96
SOT	12	3	1.11	.30–4.10	.87
Autoimmune disorder	7	3	1.96	.49–7.85	.34
AmB alone	36	18	5.77	2.66–12.5	**<.0001**
ITZ alone	67	6	0.47	.18–1.19	.113
AmB f/b ITZ	101	5	0.19	.07–0.51	**.001**

Other endemic mycoses are excluded from analysis due to zero or very small mortality counts leading to unstable OR estimates. Bold *P*-values denote statistical significance (*P* < .05).

Abbreviations: AIDS, acquired immunodeficiency syndrome; AmB, amphotericin B; CI, confidence interval; DM, diabetes mellitus; HIV, human immunodeficiency virus; ITZ, itraconazole; IVDU, intravenous drug users; OR, odds ratio; SOT, solid organ transplant.

## DISCUSSION

This systematic review represents the most comprehensive analysis to date of endemic mycoses reported from India, encompassing 893 laboratory-confirmed cases published between 1960 and 2025. The findings highlight a clear predominance of histoplasmosis and sporotrichosis, together accounting for nearly 90% of all reported cases, followed by talaromycosis as the leading systemic mycoses in India. Other geographically restricted mycoses—blastomycosis, coccidioidomycosis, emergomycosis, and paracoccidioidomycosis—remain exceedingly rare, suggesting limited endemicity, underrecognition or infrequent importation of cases from regions where these infections are more prevalent.

The wide geographic distribution of endemic mycoses across 28 Indian states and union territories challenges the traditional perception that these infections are confined to limited ecological niches. Histoplasmosis emerged as the most geographically widespread endemic mycosis, with substantial case burdens in northern and eastern India. This distribution is consistent with environmental conditions favorable to *Histoplasma* proliferation, including river basins, agricultural land, and bird or bat exposure, and parallels earlier reports from the Gangetic plains [9]. In contrast, sporotrichosis demonstrated geographic clustering in the sub-Himalayan belt, likely linked to localized environmental reservoirs, occupational exposure, and climatic conditions favoring *Sporothrix schenckii* complex [4, 10]. An earlier Indian study examined various environmental materials, including thorns, corn stalks, sphagnum moss, grass blades, and soil samples collected from areas near the homes of individuals with sporotrichosis, for the presence of *S*. *schenckii* [11]. The fungus was isolated only from soil and corn stalks, indicating that hay, corn stalks, and soil may serve as potential environmental sources of *S. schenckii* in India. Talaromycosis showed a predominant north-eastern distribution, particularly in Manipur and Assam, aligning with its established association with Southeast Asia and HIV-endemic settings. Bamboo rats (*Rhizomys sinensis*) have long been considered as enzootic reservoirs of *Talaromyces marneffei* [4]. Although the fungus has been isolated from soil in rat burrows, attempts to isolate it from other environmental sources have been unsuccessful. Consequently, the definitive environmental reservoir of *T. marneffei* remains undetermined [4]. Rare mycoses, such as blastomycosis and coccidioidomycosis, were sporadically reported, predominantly from urban centers, likely representing imported infections rather than local transmission [12, 13].

The temporal analysis revealed a pronounced rise in both the number and diversity of endemic mycoses reported after 2000, with a peak during 2011–2020. This increase was most pronounced for histoplasmosis and sporotrichosis and is likely attributable to enhanced clinical awareness, improved diagnostic availability, expansion of the medical literature, and increased reporting, rather than reflecting a true epidemiological surge alone [4, 10, 14]. In contrast, talaromycosis exhibited a more geographically and temporally restricted pattern, paralleling the emergence of HIV/AIDS and the subsequent antiretroviral roll-out in north-eastern India [4]. The emergence of emergomycosis and paracoccidioidomycosis exclusively in recent decades further supports improved recognition of previously underdiagnosed or misclassified fungal pathogens. The sporadic distribution and absence of consistent trends for blastomycosis and coccidioidomycosis underscore their rarity and potential underreporting. Nevertheless, environmental changes, urbanization, population mobility, and expanding immunocompromised populations may also be contributing to a genuine increase in disease burden.

Distinct demographic patterns were observed across endemic mycoses. The strong male predominance in histoplasmosis and talaromycosis is consistent with occupational exposure patterns and the higher prevalence of HIV and other immunocompromising conditions among men in many regions. Conversely, the female predominance seen in sporotrichosis likely reflects gender-specific occupational and domestic exposure involving soil, vegetation, and minor trauma, particularly women's involvement in horticultural and agricultural activities in rural India [10, 11]. The significant variation in age at presentation further emphasizes pathogen-specific epidemiology and host interactions, with talaromycosis and coccidioidomycosis affecting relatively younger individuals, likely due to their association with HIV and travel-related exposure, respectively.

Distinct clinical phenotypes were evident across infections. Disseminated disease predominated in histoplasmosis and talaromycosis, reflecting their propensity for systemic involvement, particularly in immunocompromised hosts. Approximately 10% of individuals with histoplasmosis develop progressive disseminated disease, which may manifest either as a chronic form characterized by oropharyngeal ulcers with or without hepatosplenomegaly or as an acute, rapidly progressive form in immunosuppressed patients [9, 15]. Although the incidence of acute progressive disseminated histoplasmosis has risen in the Asia-Pacific region alongside the AIDS epidemic, the majority of reported cases continue to represent chronic manifestations of disseminated disease [4, 9, 15]. The near-exclusive occurrence of adrenal involvement, oral lesions, and hepatosplenomegaly in histoplasmosis highlights the need to consider this diagnosis in appropriate clinical settings, particularly in TB-endemic regions, where diagnostic overlap is common. Notably, despite frequent adrenal involvement, overt adrenal insufficiency was relatively uncommon among Indian histoplasmosis cases (30/399, 7.5%), consistent with previous reports [16]. In contrast, sporotrichosis remained largely confined to lymphocutaneous and fixed cutaneous forms, with multifocal cutaneous and extracutaneous variants remaining rare. Consistent with prior studies [17, 18], cutaneous lesions were considerably more frequent in talaromycosis than in histoplasmosis, further emphasizing the divergent clinical presentations of these endemic mycoses.

Underlying host factors strongly influenced disease patterns and outcomes. We observed an overwhelming association between HIV infection and talaromycosis, consistent with global data identifying it as an AIDS-defining illness in endemic regions [19]. The association of intravenous drug use with HIV-related talaromycosis reflects evolving transmission dynamics and highlights the need for targeted screening strategies in high-risk groups. Importantly, comorbid conditions, such as type 2 diabetes mellitus and solid organ transplantation, were more frequent in histoplasmosis and coccidioidomycosis, highlighting the need for proactive surveillance in these vulnerable populations.

Diagnostic practices varied substantially by infection type and largely reflected organ involvement and available resources. Histopathology and tissue biopsy remained the cornerstone for diagnosing histoplasmosis, whereas culture positivity was highest in talaromycosis. The near absence of antigen detection and serological assays highlights a major diagnostic gap in India, likely contributing to underrecognition, misclassification, and delayed diagnosis, particularly in disseminated disease. Expanding access to rapid nonculture–based diagnostics could substantially improve early detection and outcomes [2]. Diagnosis of endemic mycoses remains a critical and persistent challenge in regions where TB is endemic [20, 21]. The present study reveals that 7.6% of endemic mycoses cases were initially misdiagnosed as TB, most notably histoplasmosis (48/399, 12%). These findings underscore the need for heightened clinical vigilance and improved access to fungal diagnostics in TB-endemic settings.

Endemic mycoses remain a significant cause of morbidity and mortality among immunocompromised populations, such as individuals with HIV, recipients of hematopoietic stem cell or solid organ transplants, patients with hematologic malignancies, and those receiving tumor necrosis factor (TNF)-α inhibitors. The increased reporting of these infections in many regions mirrors the expanding population of immunocompromised hosts [5]. Mortality among HIV/AIDS patients with disseminated histoplasmosis has been reported to range from 30% to 40% [21, 22]. Notably, in our analysis, deaths occurred predominantly among immunocompetent individuals with histoplasmosis, highlighting that severe disease and poor outcomes are not limited to traditionally high-risk groups. Instead, timely and appropriate antifungal therapy emerged as the most important determinant of survival.

Overall treatment patterns in our review were largely concordant with established guidelines. For patients with advanced HIV and moderate to severe progressive disseminated histoplasmosis, current recommendations from the European Confederation of Medical Mycology and the International Society for Human and Animal Mycology advocate initial treatment with intravenous AmB—either liposomal AmB at 3 mg/kg/day or AmB deoxycholate at 0.7–1 mg/kg/day—followed by oral ITZ at 200 mg twice daily for a minimum of 12 months [1]. In immunocompetent individuals, antifungal therapy is typically indicated for at least 6 months, with the duration tailored to the severity and site of infection [1]. In our analysis, induction therapy with AmB deoxycholate followed by oral ITZ was associated with significantly improved survival, whereas AmB monotherapy correlated with higher mortality. Although liposomal AmB has demonstrated a survival benefit over AmB deoxycholate in disseminated histoplasmosis, most cases from India were treated with the deoxycholate formulation because of limited availability and the high cost of liposomal preparations. For cutaneous sporotrichosis, SSKI has historically shown high response rates (70%–89%) [23]. Despite its proven efficacy and low cost, the use of SSKI has declined due to poor tolerability and adherence, primarily related to adverse effects, such as dysgeusia, gastrointestinal discomfort, and acneiform eruptions [23]. In our analysis, SSKI followed by oral ITZ showed high efficacy in the treatment of cutaneous sporotrichosis, supporting its continued relevance in resource-limited settings. In disseminated talaromycosis, recommended first-line therapy consists of AmB induction for 14 days followed by oral ITZ at 200 mg twice daily for 10 weeks and subsequent maintenance therapy at 200 mg daily [1]. While this regimen has been shown to improve survival, most patients in our study who received ITZ monotherapy also survived (39/41, 95%). The reliance on ITZ alone may reflect the limited availability and high cost of AmB in resource-constrained settings. In contrast, mortality remained high in coccidioidomycosis and emergomycosis, emphasizing the challenges posed by delayed diagnosis and limited therapeutic options. Collectively, these findings underscore the critical importance of early diagnosis, optimized antifungal regimens, and prompt initiation of combination therapy in severe or disseminated endemic mycoses. Although therapeutic drug monitoring was not assessed due to limited availability, its broader implementation may further improve clinical outcomes.

This review integrates 6 decades of published data and represents the most comprehensive assessment of endemic mycoses from India to date. However, several limitations warrant consideration. The evidence is derived largely from case reports and case series, which are subject to substantial publication bias, preferentially capturing unusual, severe, or disseminated cases while underrepresenting milder disease. Changes in clinical awareness, diagnostic capacity, and reporting practices since the 1960s likely contributed to the marked temporal increase observed; therefore, the number of publications should not be interpreted as a proxy for true disease incidence but rather as an increase in reported cases. Geographic distributions are also vulnerable to referral and reporting bias; major tertiary centers—particularly New Delhi, which receives referrals from across the country—may overrepresent case counts, such that apparent “hotspots” may reflect centers with better diagnostic and therapeutic facilities rather than true endemicity. Additionally, heterogeneity and missing data limited comparative and multivariate analyses, and misdiagnosis, particularly as tuberculosis, is likely underestimated. Collectively, these limitations indicate that the findings of this review are descriptive of reported cases and do not accurately reflect the true incidence, prevalence, or national burden of endemic mycoses in India.

## CONCLUSIONS

Endemic mycoses are emerging as important yet underrecognized causes of systemic fungal infections in India. Histoplasmosis and sporotrichosis constitute the majority of reported cases nationwide, while talaromycosis remains a key opportunistic infection among people with HIV, particularly in north-eastern India. The geographic distribution and clinical manifestations of these infections reflect distinct ecological, occupational, and host-related determinants. However, frequent misdiagnosis as tuberculosis, limited availability of fungal diagnostic tools, and variability in treatment practices highlight substantial gaps in current clinical management. Strengthening clinician awareness; expanding access to fungal diagnostics, especially serological and molecular methods; and implementing structured national surveillance systems are critical priorities. Designating endemic mycoses as notifiable diseases may further enhance early detection and improve patient outcomes. Overall, these findings highlight an evolving landscape of endemic mycoses in India that warrants focused public health action and stronger integration of medical mycology into the national infectious disease framework.

## Supplementary Material

ofag171_Supplementary_Data

## Supplementary Data

Supplementary materials are available at *Open Forum Infectious Diseases* online. Consisting of data provided by the authors to benefit the reader, the posted materials are not copyedited and are the sole responsibility of the authors, so questions or comments should be addressed to the corresponding author.
